# Evaluation of Physiological Activity of Long-Term Ripened Gouda Water Extract

**DOI:** 10.3390/foods13213446

**Published:** 2024-10-29

**Authors:** Woojin Ki, Gereltuya Renchinkhand, Hyoungchurl Bae, Myoung Soo Nam

**Affiliations:** 1 Department of Dairy Science, College of Agriculture & Life Sciences, Chungnam National University, Daejeon 34134, Republic of Korea; rl8917@naver.com (W.K.); hcbae@cnu.ac.kr (H.B.); 2 Department of Biology, School of Arts and Sciences, National University of Mongolia, Ulaanbaatar 14201, Mongolia; handgai@yahoo.com

**Keywords:** ripened Gouda, bioactive peptides, antioxidant, ACE inhibition, anti-inflammatory activity

## Abstract

This study investigated peptide changes and their bioactive functions through the long-term ripening of Gouda. Young Gouda (YG), medium Gouda (MG), and extra-sharp Gouda (EG) water extracts were prepared and functional peptides were recognized using liquid chromatography–high-resolution mass spectrometry. Two peptides with ACE-inhibitory effects (IQP and LQP) were identified in YG, while in MG and EG were identified eight (EL, IVP, VP, LPP, VIP, IPP, VPP, and VVPP) and six (EL, YL, VP, IR, YPEL, and DKIHPF) functional peptides, respectively. MG (70.26%) and EG (46.81%) showed stronger antioxidant activity than YG (25.99%) in ABTS (2,2′-azino-bis-(3-ethylbenzothiazoline-6-sulfonic) acid) inhibition, though the DPPH (2,2-diphenyl-1-picrylhydrazyl) inhibition rate decreased with ripening. The antihypertensive effect increased in MG (79.76%) and EG (94.50%) due to ACE-inhibitory peptides. Measurements of inflammatory mRNA expression levels and immunoblotting were conducted to assess the anti-inflammatory properties. MG and EG suppressed the transcription of IL-1β and IL-6 mRNA. Immunoblotting indicated that EG suppressed IκBα phosphorylation to 57%. The enhancement of bioactive function in the water-soluble part of long-term ripened Gouda cheese may have affected identified peptides as well as unknown peptides. Further studies are expected to aid in discovering these novel bioactive peptides.

## 1. Introduction

Bovine milk is a complete food, containing almost all the nutrients that humans need, and it is highly digestible. This food contains approximately 3.4% protein, including biologically active caseins and functional proteins (lactoferrin, transferrin, immunoglobulin, etc.). Milk protein is highly nutritious and is currently recognized as the origin of peptides with significant physiological activity [1].

Bioactive peptides are protein fractions that play diverse roles in physiological functions and are beneficial for physical and mental health [2]. Peptides are involved in the cellular responses within the body by signaling biological processes, transporting trace elements, and inhibiting neurotransmitters and enzymes [3]. The physiological function of peptides is rooted in the types and arrangement of amino acids [4]. Additionally, the size of the peptide sequence also has an effect, and the size of multifunctional peptide sequences has been seen to range from 2 to 20 amino-acid arrangements [3]. These peptides exhibit antioxidant, antihypertensive, anti-thrombotic, and immunomodulatory effects, and they have received significant interest owing to their possible biological benefits [5]. Milk protein-derived peptides can be obtained from popular sources of animal protein like yogurt, cheese, and kefir. Moreover, bioactive peptides have been confirmed to positively impact metabolic diseases like hypertension, obesity, and hyperlipidemia [6].

Cheese is a long-standing food that has been made in human society for millennia, with more than 1000 types of cheese currently available [7]. Cheese is a popular dairy food that, depending on the type, is appreciated for its unique taste, pleasant texture, and nutrient-rich content. Cheese is rich in protein and features numerous peptides derived from milk. The level of protein breakdown and the different processing techniques applied during production and aging determine the composition of these elements [8]. Milk protein degradation is affected by various elements, including pH, chymosin, enzymes produced by microbes, salt, storage duration, temperature, and humidity [9].

Enzymes in rennet or produced by microbes can decompose the casein in milk, leading to the formation of peptides with physiological functions [9]. In particular, during the ripening process, numerous fractions emerge from the caseins (αS1-, αS2-, β-, κ-casein), mainly via cheese starters and foreign microbial-derived proteolytic enzymes [10]. Therefore, even two different samples of the same type of cheese are likely to have bioactive peptides that vary in type, amount, and function, depending on the degree of ripeness.

Gouda cheese originated in the town of Gouda, located between Rotterdam and Utrecht in southern Poland in the western Netherlands, and has been produced since the 6th century. Gouda is a round-wheeled, semi-hard type of cheese that is coated with yellow-orange wax and aged for short or long periods to extend its shelf life [11]. Gouda cheese’s physiologically active properties change significantly depending on the ripening stage. A study of Gouda cheese’s antioxidant and ACE (Angiotensin Converting Enzyme)-inhibitory effects [12] showed that the peptides present in the cheese, as well as their bioactivity, continuously change depending on the ripening stage.

Therefore, studying the diversity and physiological role of peptides in Gouda cheese at different ripening periods will likely uncover changes in composition and function and reconfirm their value as fermented foods. This study investigated the potential of long-ripened Gouda cheese as a health food by evaluating its antioxidant, immunomodulatory, and antihypertensive activity at various stages of ripening.

## 2. Materials and Methods

### 2.1. Gouda Samples and Manufacturing Gouda Water Extracts (WEs)

The Gouda used in the study was classified into EG (extra-aged Gouda), MG (medium Gouda), and YG (young Gouda) groups, and samples of extra-sharp (3-years ripened), medium (6-months ripened), and young (less than 1-month ripened) Gouda, produced and ripened at the Chungnam National University Animal Resources Research Center, were used. Each Gouda was evenly ground, and 180 mL of sterilized distilled water (DW) was blended with 20 g of Gouda. The mixture was extracted at 40 °C for 1 h using an ultrasonic extractor (POWERSONIC 420, HWASHIN, Seoul, Republic of Korea). After cooling, WE was divided in layers by centrifugation (Supra R17, Hanil, Gimpo, Republic of Korea) at 6000× *g* for 20 min at 4 °C. Then, the cooled lipid layer was taken off and the supernatant was collected and sieved through Whatman No. 2 (Whatman, Maidstone, UK). In addition, because long-term ripened Gouda contains decomposition products of various sizes and has a high content of amino acids and short peptides, filtration according to molecular weight was not performed to fully evaluate the physiological activity of the extract. The obtained WE was freeze-dried and stored at −20 °C.

Samples for mass spectrometry were modified from the method used in cheese peptide determination [13]. The Gouda WEs extracted as described above were freeze-dried and dissolved in deionized water (100 ppm).

### 2.2. Determination of Water-Soluble Nitrogen in Gouda

As an indicator of cheese ripeness, the quantification of nitrogen compounds was carried out to observe variations of the nitrogen content in the water-soluble part.

The Gouda was finely ground, and 20 mL of sterilized DW was added to 5 g of the Gouda. The mixture was homogenized for 5 min using an ULTRA TURRAX T25 (IKA Co., Wilmington, NC, USA). It underwent centrifugation and was sieved, as described in manufacturing Gouda WEs. This filtrate was used in the experiment below.

Following Hull’s method [14], 2.5 mL of the filtrate was mixed with 5.0 mL of reagent A (12% trichloroacetic-acid aqueous solution). After leaving the mixture at room temperature for 20 min, it was filtered through Whatman No. 42 (Whatman, Maidstone, UK). Then, 2.5 mL of the filtrate was taken, and 5.0 mL of reagent B (75 g of sodium carbonate and 10 g of hexametaphosphate dissolved in 500 mL of DW) and 1.5 mL of reagent C (50 mL of Folin and Ciocalteu’s phenol (Sigma Aldrich, Saint Louis, MO, USA) mixed with 100 mL of DW) were added and reacted in a heating bath at 30 °C for 30 min. After the reaction was completed, its optical density (OD) was measured at 570 nm with SpectraMax ABS Plus (Molecular Devices, San Jose, CA, USA). The WSN content was calculated by Equation (1) with tyrosine as the standard (0, 20, 40, 80 μg).

(1) y=0.0073x−0.0012

R^2^ = 0.9989; *y*, OD at 570 nm; *x*, tyrosine content (μg).

### 2.3. Measurement of Protein and pH in Extracts

Protein and pH assessments were conducted to verify the alterations in the properties of Gouda WEs. Protein concentrations of Gouda WE were measured using Bradford’s technique [15]. The Gouda WE powder was dissolved in sterile DW (10 mg/mL) for measurement. The dye solution was prepared by diluting dye reagent concentrate (Bio-rad, Hercules, CA, USA) with DW at a 1:4 ratio. The measurement was performed by adding 10 μL of the Gouda WE solution and 200 μL of the dye solution to a microplate (96-well), and the OD was then read at 595 nm with SpectraMax ABS Plus (Molecular Devices, San Jose, CA, USA). After that, bovine serum albumin (BSA, Bio-rad, Hercules, CA, USA) was used as a standard and expressed as BSA equivalent.

The alterations in pH were assessed at 25 °C using an S-20K (Mettler Toledo Co., Columbus, OH, USA) following the combination of each freeze-dried WE (YG, MG, and EG) with DW at a 2% (*w*/*v*) concentration.

### 2.4. Mass-Spectrometry Analysis of Gouda WEs

The functional peptides of Gouda WE were identified by LC-HRMS (ultra-liquid chromatography–high-resolution mass spectrometry) analysis. The UHPLC system (1290 Infinity II) was combined with a high-resolution mass spectrometer (TripleTOF 5600 plus) equipped with Electrospray ionization (Duospray), both manufactured by SCIEX, Framingham, MA, USA.

The Gouda WE solution (100 ppm) was filtered through a 0.2 μm syringe filter (hydrophilic PTFE, Hyundai Micro, Anseong, Republic of Korea). Twenty μL of WE solution was injected and separated by passing through the column with a two-solvent gradient elution. The operating time was 15 min, and the mobile phases used were A: deionized water (*v*/*v*) with 0.1% formic acid, and B: acetonitrile (*v*/*v*) with 0.1% formic acid. The ratio (A:B) of mobile phase started at 9:1 and ended at 1:9.

The components separated by the UHPLC system were ionized by electrospray and analyzed by mass spectrometry. Mass spectrometry was performed in (+) ion mode. The instrument conditions were as follows: flow rate, 0.6 μL; source temperature, 100 °C; collision energy, 35 eV; desolvation gas flow, 30 L/h. Nitrogen gas was used. For mass spectrometry, functional peptide information was collected from the Milk Bioactive Peptide Database (MBPDB) [16] in advance, and the chemical formula of each peptide was calculated. Based on this, expected *m*/*z* values were determined. Analyst TF 1.8.1 (SCIEX, Framingham, MA, USA) software was used for the interpretation of mass-spectrometry results. The recorded mass spectrum was analyzed to confirm the chemical structure, chemical reactions, and molecular weight. Functional peptides were identified by comparing the measured formula and *m*/*z* ratio based on the formula and predicted m/z ratio of the previously investigated peptide.

### 2.5. Antioxidant Effect—2,2’-Azinobis-(3-Ethylbenzothiazoline-6-Sulfonic Acid)

The 2,2’-azinobis-(3-ethylbenzothiazoline-6-sulfonic acid) (ABTS) inhibition ability of the Gouda WE was confirmed through decolorization [17]. The ABTS working solution was created by mixing a 7 mM ABTS (Sigma-Aldrich, Saint Louis, MO, USA) with a 2.4 mM potassium persulfate. The working solution was then stored without light at 25 °C for 12~16 h before the experiment. The ABTS working solution was diluted with DW until the OD measured at 734 nm reached 0.7 (±0.02). A mixture was prepared by combining 50 μL of Gouda WE solution (concentration of 5 mg/mL in distilled water), with 950 μL of ABTS working solution. After a 10 min reaction, the OD at 734 nm was recorded using a SpectraMax ABS Plus (Molecular Devices, San Jose, CA, USA). As a positive control, L-Ascorbic acid (25, 50 μg/mL) was used. The inhibition rate was determined using Equation (2). In Equation (2), the control was obtained by reacting distilled water with the ABTS working solution instead of the sample (Gouda WE solution), and each blank was treated with distilled water instead of the ABTS working solution.

(2) $$\mathrm{Inhibition}\mathrm{Rate}\;(\%)=\frac{1-\left(sample-sample\;blank\right)}{control-control\;blank}\times 100$$

### 2.6. Antioxidant Effect—DPPH

The antioxidant evaluation method [18] confirmed the restraint of 2,2-diphenyl-1-picrylhydrazyl (DPPH) by Gouda. A total of 20 μL of Gouda WE solution was added to a microplate (96-well) that contained 180 μL of freshly made 0.2 mM 2,2-diphenyl-1-picrylhydrazyl (Sigma-Aldrich, Saint Louis, MO, USA) solution in methanol. After reacting in the darkroom for 15 min, the change in OD at 517 nm was measured with SpectraMax ABS Plus (Molecular Devices, San Jose, CA, USA). The OD for blank was determined in the same way using DW instead. The inhibition rate was determined using Equation (3).

(3) $$\mathrm{Inhibition}\mathrm{Rate}\;(\%)=\left[1-\frac{S}{S_{0}}\right]\times 100$$

*S*, the OD of the Gouda WE; *S*_0_, the OD of the DW.

### 2.7. Antihypertensive Activity: ACE Inhibition

ACE inhibition was assessed using a modified spectrophotometric analysis technique in vitro [19]. Rabbit-lung acetone powder (Sigma-Aldrich, Saint Louis, MO, USA) was combined in a 1:10 ratio with a 0.1 M borate buffer solution containing 0.3 M NaCl. This mixture was extracted by stirring at 4 °C for 24 h. The enzyme solution was obtained by collecting the supernatant following centrifugation at 15,000× *g* for 30 min. Hippuryl-histidyl-leucine (HHL, Sigma Aldrich, Saint Louis, MO, USA) was prepared at a 5 mM concentration by diluting it in a 0.1 M sodium borate buffer with 0.3 M NaCl, then utilized as a substrate. Captopril (Sigma-Aldrich, Saint Louis, MO, USA) was chosen as a positive control due to its excellent inhibitory activity. Next, a mixture of 100 μL volume of a 0.1 M sodium borate buffer and 50 μL of enzyme solution was gently added to the 50 μL sample, which had been pre-incubated at 37 °C for 5 min. Subsequently, a 50 μL portion of the substrate was introduced, and the reaction proceeded at 37 °C for 30 min. Enzyme activity was halted by adding 0.1 N HCl (300 μL). Following this, 1 mL of ethyl acetate was added and vortexed for 15 s. It was then centrifuged at 960× *g* for 5 min at a temperature of 4 °C. The supernatant was gathered and dried using a Modulspin 31 (Hanil, Daejeon, Republic of Korea), and then DW was added (1 mL) and dissolved well. The OD at 228 nm was assessed using the SpectraMax ABS Plus (Molecular Devices, San Jose, CA, USA).

To prepare the sample blank, the reaction was halted using 0.1 N HCl (300 μL) before the addition of the enzyme solution. Inhibition (%) was produced using Equation (1).

### 2.8. Immunomodulatory Function: Anti-Inflammation

#### 2.8.1. Cell Cultivation

The experiment employed RAW 264.7, which was provided by the Korean Cell Line Bank. Cells were kept at 37 °C in a 5% CO_2_. The medium utilized was Dulbecco’s modified Eagle’s medium (DMEM, WELGENE, Gyeongsan, Republic of Korea), and it was enhanced with 10% (*v*/*v*) FBS (fetal bovine serum, WELGENE, Gyeongsan, Republic of Korea) and 1% (*v*/*v*) penicillin and streptomycin (WELGENE, Gyeongsan, Republic of Korea).

#### 2.8.2. Cytotoxicity Evaluation

We conducted a cell proliferation assay [20] to confirm the influence of Gouda WE on the survival of RAW 264.7. RAW 264.7 cells were plated at a density of 3000 cells/well in a microplate (96-well) and incubated for 24 h. Different concentrations of samples were treated in each well and additionally cultured for 24 h. Next, the MTS (3-(4,5-dimethylthiazol-2-yl)-5-(3-carboxymethoxyphenyl)-2-(4-sulfophenyl)-2H-tetrazolium salt) solution (Promega, Madison, WI, USA) was added to each well and OD readings were taken (490 nm) with a SpectraMax ABS Plus (Molecular Devices, San Jose, CA, USA). The cytotoxicity of each Gouda WE was assessed by calculating it as a percentage relative to the activity observed in the untreated control group.

#### 2.8.3. Quantitative Real-Time PCR

RAW 264.7 cells were seeded in a 6-well format at a concentration of 1 × 10^5^ cells/well and allowed to incubate for 24 h. After that, the culture medium was substituted with one containing 100 μg/mL of Gouda WE and cultivated for 24 h. Subsequently, cells were placed for 6 h in the presence of 1 μg/mL lipopolysaccharide (LPS). The medium was eliminated, cleaned with cold PBS, and the cells were processed with Ribo Ex (Gene All, Seoul, Republic of Korea). Purified RNA was obtained using the Hybrid-R RNA purification kit (Gene All, Seoul, Republic of Korea). To synthesize cDNA, RNA was quantified with the Nabi UV/Vis Nano spectrophotometer (Micro Digital, Seongnam, Republic of Korea). To the 1 μg of RNA, we added 1 μL of random hexamer (100 pmol/μL) and 1 μL of a 10 mM dNTP mixture. The overall volume was then adjusted to 10 μL using DEPC (Diethyl pyrocarbonate)-treated water. Subsequently, we incorporated an M-MLV reverse transcriptase (1 μL; Promega, Madison, WI, USA), a 5X M-MLV RT reaction buffer (4 μL; Promega, Madison, WI, USA), an RNase inhibitor (1 μL; Enzynomics, Daejeon, Republic of Korea), and DEPC-treated water (4 μL). The mixture was kept at 25 °C for 10 min followed by incubation at 50 °C for 1 h to enable cDNA synthesis.

The expression of immunomodulatory cytokines was compared by quantitative real-time polymerase chain reaction (qRT PCR) using an AriaMx (Agilent, Santa Clara, CA, USA). The reaction was carried out under these conditions: denaturation at 95 °C for 20 s, annealing at 58 °C for 20 s, and extension at 72 °C for 20 s, repeated for 40 cycles. Table 1 shows the primer sequences used in the chain reaction.

#### 2.8.4. Immunoblotting

As performed in 2.8.3, RAW 264.7 was cultured in a 6-well format at 1.5 × 10^5^ cells/well for 24 h. After that, cells were treated with each Gouda WE (100 μg WE in 1 mL culture medium) and cultured for 24 h. To induce an inflammatory response, LPS was applied at a dose of 1 μg/mL for 30 min. Immediately after, the medium in each well was rinsed with PBS. Cell lysates were obtained using a lysis buffer composed of 10 mM Tris-HCl (pH 7.5), 100 mM NaCl, 1 mM EDTA, 10% glycerol, and 1% Triton X-100. The solution underwent sodium dodecyl sulfate-polyacrylamide gel electrophoresis (SDS-PAGE) for one hour, followed by a transfer to a nitrocellulose membrane. Blocking was subsequently carried out with 5% non-fat dry milk in PBST for one hour. The membrane was subjected to antibodies specific to p-I κBα (1:1000), I κBα (1:1000), and GAPDH (1:2000), all from Cell Signaling Technology, Danvers, MA, USA, for a duration of 12 h. Following the PBST wash, the membrane was reacted with a horseradish peroxidase-conjugated secondary antibody (Cell Signaling Technology, Danvers, MA, USA) for 1 h at 25 °C. Membranes were utilized with Supersignal (Thermo Fisher Scientific, Waltham, MA, USA) and luminescence was identified with Luminograph I (ATTO, Tokyo, Japan). Photographed signals were edited and calculated through ImageJ 1.8.0_172 software (NIH, Bethesda, MD, USA).

### 2.9. Statistical Processing

Each experiment was conducted in technical triplicate analysis (three measurements of the same extract obtained from a few grams of a single cheese), and results were shown as an average ± standard error. The dataset was processed by analysis of variance (ANOVA), and the averages were confirmed with the Tukey test, applying a significance level of either 1% or 5%. A paired *t*-test was utilized to examine the cytotoxicity.

## 3. Results and Discussion

### 3.1. Variations in WSN of GUDA

The changes in WSN show that WSN increased with the ripening but decreased after long-term ripening (Table 2).

During the ripening period, the rise in WSN of cheese is usually attributed to casein degradation, which can differ based on the proteases originating from the rennet or starter used in its manufacture [9]. Moreover, it has been established that the protease generated by the starter leads to ongoing breakdown [21]. Thus, the rise in water-soluble nitrogens serves as a marker for the progression of ripening.

In this study, WSN increased in medium Gouda but decreased in extra-sharp Gouda compared to medium Gouda. This indicates that proteolysis occurred rapidly in the early stages of ripening. Also, it shows that microbial metabolism and enzymatic action led to a decline in the total amount of WSN during the long ripening period of three years.

### 3.2. Protein Quantification and pH of Gouda WEs

Table 3 presents the protein concentrations and pH values for each Gouda WE. Each Gouda WE powder was entirely dissolved in distilled water at a specific concentration for this analysis.

The protein measurement results were 20.01 mg BSAE/g DM for YG and 20.12 mg BSAE/g DM, for MG, showing no notable difference, but that of EG decreased to 12.09 mg BSAE/g DM. This appears to be due to the breakdown of proteins into small peptides and an increase in other metabolites. The pH of Gouda WE significantly increased from YG (5.69) to MG (6.01) but showed no significant difference in EG (5.72).

### 3.3. Identification of Bioactive Peptides of Gouda WEs

**Figure 1:**
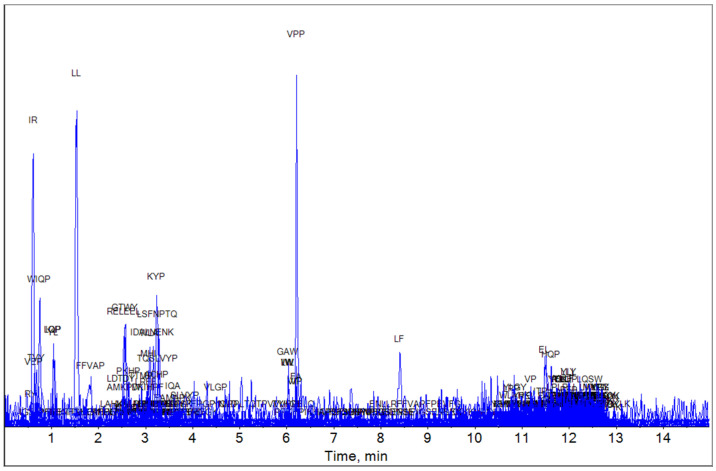
Detection of young Gouda water extract by mass spectrometry. The figure shows matched peptides and all peaks observed during elution.

**Figure 2:**
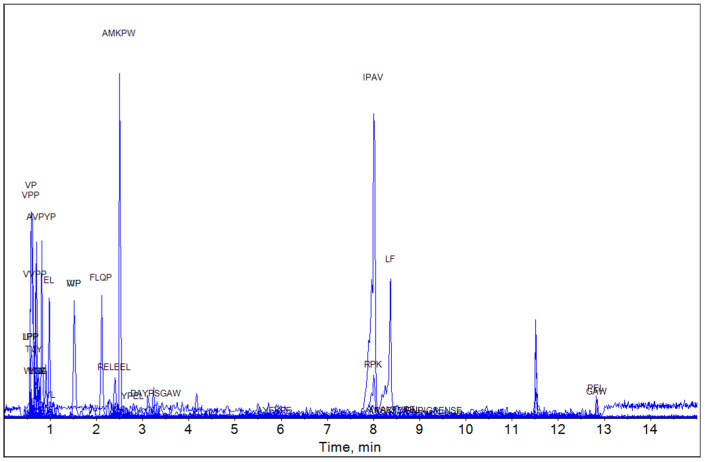
Detection of medium Gouda water extract by mass spectrometry. The figure shows matched peptides and all peaks observed during elution.

**Figure 3:**
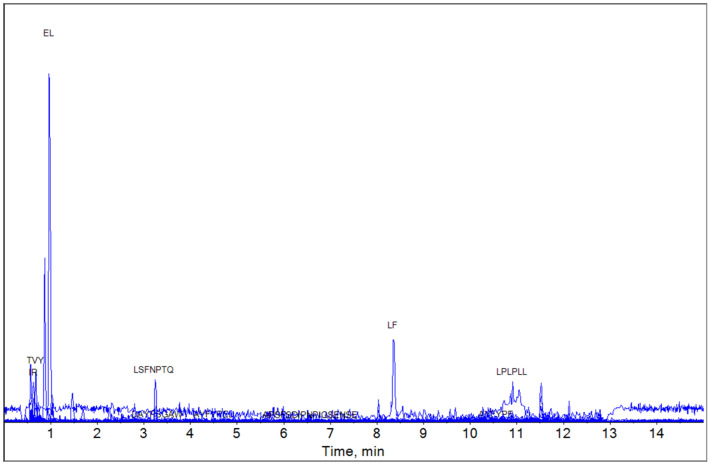
Detection of extra-sharp Gouda water extract by mass spectrometry. The figure shows matched peptides and all peaks observed during elution.

Based on the MBPDB data, the bioactive peptide compositions of YG, MG, and EG were analyzed using LC-HRMS. The LC-HRMS results for YG, MG, and EG are shown in Figure 1, Figure 2 and Figure 3, respectively.

The peptides that exceeded the set threshold (1000) along with their known physiological activity are shown in Table 2, Table 3 and Table 4. IQP and LQP (which have ACE-inhibitory function) were found based on matching from the database (Table 4).

In the case of MG (aged for 6 months) several peptides with notable biological activities were identified. EL is known to have antioxidant activity. IVP, VP, LPP, VIP, and VVIP have the effect of inhibiting ACE, while IPP and VPP are recognized for their multifunctional properties, including immunomodulatory, antioxidant, and ACE inhibition (Table 5).

In EG (3-year ripening), some peptides disappeared with long proteolytic periods, and new physiologically active peptides (YL, IR, YPEL, and DKIHPF) were detected (Table 6).

According to our findings, we discovered peptides with antihypertensive potential in YG and several peptides with antioxidant, immunomodulatory, and antihypertensive potential in MG. Similarly, we identified peptides with antioxidant and ACE-inhibitory properties in EG. Among the three experimental groups, the MG contained the most diverse types of functional peptides. In addition, it has been observed that different types were produced at each ripening stage. These results confirm that various bioactive peptides are produced owing to the breakdown of proteins during the ripening process, and that the peptide composition changes over time.

### 3.4. ABTS Inhibition of Gouda WEs

**Figure 4:**
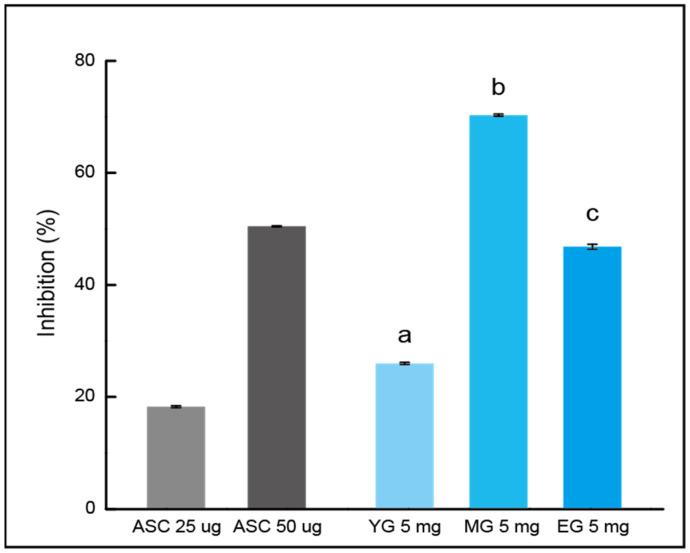
Inhibitory effect on the 2,2’-azino-bis (3-ethylbenzothiazoline-6-sulfonic acid) by water extract of Gouda. Each result is shown as the mean and standard error (3 repetitions). Matching letters exhibit no significant difference (*p* < 0.01). ASC, L-ascorbic acid; YG, young Gouda extract; MG, medium Gouda extract; EG, extra-sharp Gouda extract.

The inhibitory effect on ABTS radicals was conducted by treating each sample with ABTS solution, as shown in Figure 4. YG showed a radical inhibition rate of 25.99 ± 0.18%. The scavenging activity increased to 70.26 ± 0.18% due to the change caused by ripening in MG. However, the scavenging activity for EG was 46.81 ± 0.44%, which was higher than that of YG but lower than that of MG. The decrease observed after the initial increase aligned with findings from earlier research [32], which evaluated antioxidant activity according to the ripening stage of Cheddar cheese.

### 3.5. DPPH Inhibition of Gouda WEs

**Figure 5:**
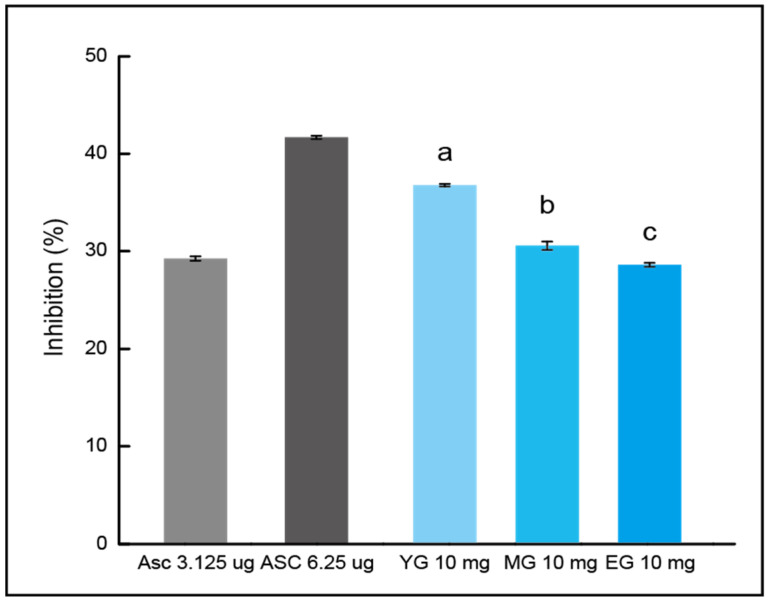
Inhibitory effect on the 2,2-diphenyl-1-picrylhydrazyl by water extract of Gouda. Each result is shown as the mean and standard error (3 repetitions). Matching letters exhibit no significant difference (*p* < 0.01). ASC, L-ascorbic acid; YG, young Gouda extract; MG, medium Gouda extract; EG, extra-sharp Gouda extract.

The inhibitory effect of Gouda on DPPH gradually decreased during the ripening, as displayed in Figure 5. At the first part (YG), the inhibition rate was 36.77 ± 0.14%. In MG, it was 30.58 ± 0.42%, and for EG (ripened for 3 years) it was 28.60 ± 0.20%. Contrary to the findings from ABTS, the inhibitory effect on DPPH was not increased by ripening and was the lowest in EG. Ripening of Gouda did not positively affect the DPPH radical scavenging, and as observed in the previous ABTS data, the activity tended to decline as the ripening proceeded.

The results of the inhibition of the DPPH radical and the ABTS assay suggest that Gouda cheese is expected to show the best antioxidant activity when it undergoes an appropriate level of ripening rather than long-term ripening. There is a need for further research to compare the antioxidant activity by subdividing the ripening stages of Gouda. However, while the activity decreased in DPPH, the ABTS radical inhibition was still significantly higher compared to the early stage of ripening. The value of long-term ripened Gouda was positively evaluated in light of this, along with other functionalities.

### 3.6. ACE Inhibition Potential

The ability of Gouda cheese WE to lower high blood pressure was assessed by examining its ability to inhibit the activation of ACE. The ACE converts angiotensin I into angiotensin II. This conversion simultaneously deactivates bradykinin, a vasodilator, thereby producing a potent vasoconstrictor effect and increasing blood pressure [27].

In the present study, ACE inhibition increased with longer ripening periods (Table 7). As a raw Gouda, YG demonstrated relatively low ACE-inhibitory activity, measured at 47.02%. In contrast, MG and EG presented significantly high ACE inhibition, which was related with the emergence of various peptides with ACE inhibition that have been previously analyzed. MG showed a high inhibition rate of 79.76%, whereas EG showed the highest inhibition rate with an increase of 94.50%.

Results from previous studies on the peptides of ripened cheese [33] have reported that ACE suppression function depends on the extent of proteolysis, and that this ability gradually decreases as a result of extensive proteolysis after peaking. The study results indicate that inhibitory ability tends to enhance with ripening. However, the ACE-inhibitory ability of Gouda did not decrease after 6 months (medium) and was stronger at 3 years (extra-sharp). Additionally, goat cheese shows higher ACE-inhibitory activity compared to cheeses like Emmental, Camembert, and Edam [34]. It is expected that, with long-term ripening, it could be noted for its excellent functionality in addition to flavor compared to other cheeses.

### 3.7. Anti-Inflammatory Effects of Gouda WEs

#### 3.7.1. Effects of Gouda WEs on Cell Viability

Macrophages were exposed to YG, MG, and EG, and cell proliferation analysis was performed to assess the cytotoxicity of the samples. The concentration range was 500 to 50 μg/mL, and each group was cultured for 24 h after treatment. The cytotoxicity of the sample was evaluated by comparing the cell viability with that of the untreated group.

**Figure 6:**
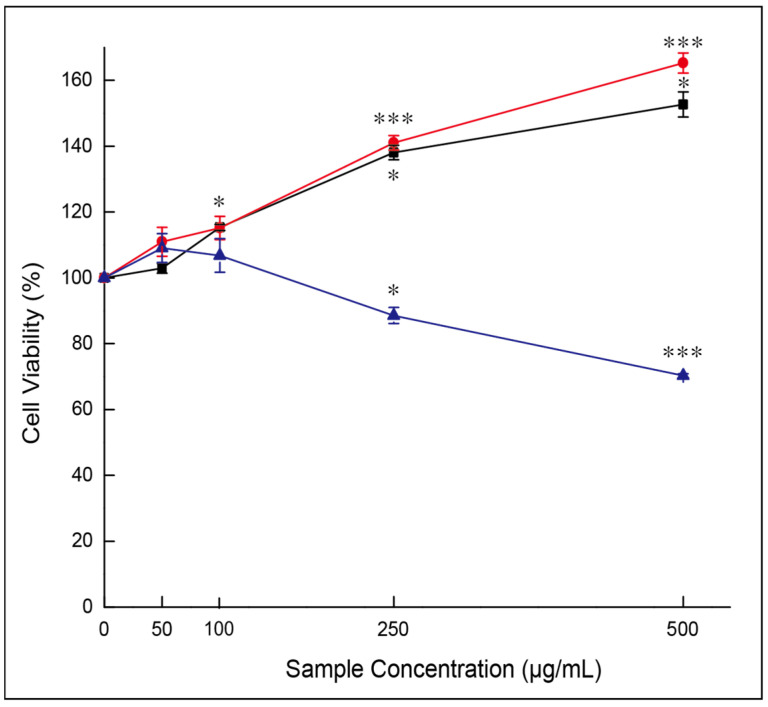
Effects of Gouda water extracts on the survival of RAW 264.7 cells. Each result is shown as the mean and standard error (3 repetitions). *, difference from the nontreated group at 5% significance level; ***, difference from the nontreated group at 0.1% significance level (paired *t*-test). ■, YG (young Gouda extract); ●, MG (medium Gouda extract); ▲, EG (extra-sharp Gouda extract).

Cell viability in the YG- and MG-treated groups at all concentrations was higher than control, whereas EG showed lower cell viability at high concentrations (Figure 6). When samples were treated for 500 μg/mL, the cell survival rate was 152.65 ± 3.80% for YG, 165.25 ± 3.03% for MG, and 70.34 ± 0.57% for EG. The impact of the sample was lessened as the concentration decreased, and no cytotoxicity was found at 100 μg/mL, with YG showing 115.32 ±0.90%, MG 115.16 ± 3.53%, and EG 106.81 ± 5.12%.

As a result, the treatment concentration of Gouda WEs to RAW 264.7 was established at 100 μg/mL, as it did not notably impact cell viability.

#### 3.7.2. Change in Inflammatory mRNA Levels

**Figure 7:**
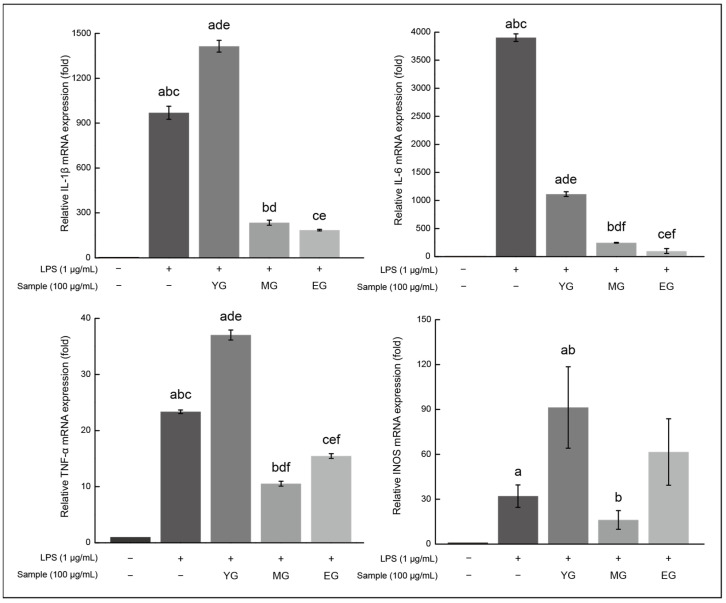
Impact of Gouda WEs on the transcription of inflammatory mRNA. Each result is shown as the mean and standard error (3 repetitions). Matching letters above each bar suggest no significant difference (*p* < 0.05). WE, water extract; LPS, lipopolysaccharide; IL-6, interleukin-6; IL-1β, interleukin-1β; TNF-α, tumor necrosis factor-α; iNOS, inducible nitric oxide synthase; YG, young Gouda extract; MG, medium Gouda extract; EG, extra-sharp Gouda extract.

IκBα phosphorylation triggers the activation of NF-κB, resulting in the secretion of inflammatory mediators like IL-6, IL-1β, TNF-α, and iNOS [35]. To evaluate the influence of Gouda WE on the LPS-induced inflammation in macrophages, changes in mRNA transcription were compared in Figure 7.

The expressions of inflammation related to mRNA, including IL-1β and IL-6, decreased with maturation. Specifically, IL-1β expression decreased to 2.36% and IL-6 expression decreased to 19.04% in the EG treatment group. On the other hand, TNF-α expression decreased to 66.20% compared to the TNF-α expression of the control, but it was slightly higher than that of MG, which decreased to 45.06%. We found that iNOS expression increased by 187.25% compared with that in the control plot. Regarding the functional peptide profile in this study, it appears that the immunomodulatory peptides produced during Gouda ripening contributed to the noted anti-inflammatory properties. As a result, not all mRNA levels decreased, but IL-1β and IL-6 levels reduced, with the effect becoming more evident as ripening advanced.

Contrary to the fact that YG increased the immune response of macrophage, both MG and EG significantly inhibited mRNA expression excluding iNOS, which is not unrelated to the appearance of immunomodulatory peptides. These results seem to be influenced by the emergence of peptides such as IPP and VPP in MG. Additionally, although no anti-inflammatory peptides were identified in the case of EG, it may be due to the influence of previously unknown peptides, and interesting results may be obtained through further detailed analysis.

#### 3.7.3. Suppression of IκBα Phosphorylation

**Figure 8:**
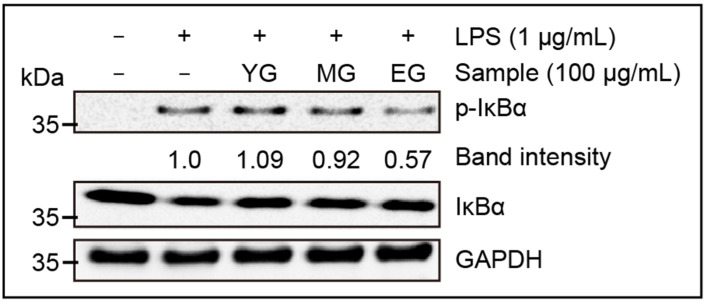
Suppression of IκBα phosphorylation by Gouda WE. LPS, lipopolysaccharide; GAPDH, Glyceraldehyde-3-phosphate dehydrogenase; IκBα, nuclear factor of κ light polypeptide gene enhancer in B-cells inhibitor α; WE, water extract; YG, young Gouda extract; MG, medium Gouda extract; EG, extra-sharp Gouda extract.

Our research showed that properly matured Gouda WE effectively inhibited IL-1β and IL-6 signaling in the macrophage cell line. Based on this fact, it was hypothesized that Gouda WE would inhibit the phosphorylation level of IκBα in the NF-κB pathway that induces inflammation. Accordingly, we performed an immunoblot of IκBα and p-IκBα to determine its effect on NF-κB pathway activity, which primarily regulates inflammatory responses. Figure 8 demonstrates that YG caused IκBα phosphorylation 9% more than LPS alone. On the other hand, MG reduced the LPS-triggered phosphorylation of IκBα to 92%. Furthermore, in EG treatment, IκBα phosphorylation was inhibited to 57% and exhibited strong anti-inflammatory effects. From these results, inhibitory activity was not evident in YG, but it seems to inhibit the inflammation by reducing the phosphorylation of IκBα by peptides formed during the ripening period in MG and EG.

We confirmed that extra-sharp Gouda WE effectively inhibited inflammatory responses both in the nucleus and in the cytoplasm based on its impact on inflammatory mRNA expression and IκBα phosphorylation. Therefore, long-term ripened Gouda could create high value as an anti-inflammatory functional food. However, as mentioned earlier, a detailed analysis of the undiscovered peptides must be conducted first.

## 4. Conclusions

In this study, water extracts of young, medium, and extra-sharp Gouda were analyzed for their bioactive peptide composition and various health properties. The study found that different ripening periods resulted in the emergence of different bioactive peptides in each ripening stage. The activity of Gouda WE as an antioxidant was evaluated, and long-term ripening was not positive for antioxidant activity. In terms of antihypertensive properties, it was shown that ACE inhibition increases with ripening. In particular, the anti-inflammatory effects revealed extra-sharp Gouda effectively suppresses inflammatory responses at both gene-expression and protein levels. These findings suggest that long-term ripened Gouda can be valued as a functional food that may offer health benefits related to high blood pressure and inflammation. This study did not perform peptide purification and quantification of the extract. Further analysis and purification of the peptides, as well as biological experiments to support the research findings, need to be conducted. Furthermore, due to the limitations of the scale of this study, the reproducibility among multiple cheeses with the same stage could not be confirmed. Research on this is necessary to support the reliability of the results of this experiment.

## Figures and Tables

**Table 1:** Primer sequence information of each target mRNA.

Target	Primer (Forward)	Primer (Reverse)
IL-1β	5’-AGG TCA AAG GTT TGG AAG CA-3’	5’-TGA AGC AGC TAT GGC AAC TG-3’
IL-6	5’-GTC CTT CAG AGA GAT ACA GAA ACT-3’	5’-AGC TTA TCT GTT AGG AGA GCA TTG-3’
TNF-α	5’-AGG GTC TGG GCC ATA GAA CT-3’	5’-CCA CCA CGC TCT TCT GTC TAC-3’
iNOS	5’-CAG CTG GGC TGT ACA AAC CTT-3’	5’-CAT TGG AAG TGA AGC GTT TCG-3’
GAPDH	5’-CCA TGG AGA AGG CTG GGG-3’	5’-CAA AGT TGT CAT GGA TGA CC-3’

Note: IL-6, interleukin-6; IL-1β, interleukin-1β; TNF-α, tumor necrosis factor-α; iNOS, inducible nitric oxide synthase; GAPDH, Glyceraldehyde-3-phosphate dehydrogenase.

**Table 2:** Variations in WSN during the ripening of Gouda.

Sample	WSN (mg Tyrosine/kg)
Young Gouda	20.74 ± 0.19 ^a^
Medium Gouda	149.24 ± 0.57 ^b^
Extra-sharp Gouda	69.73 ± 0.58 ^c^

Note: Each result is presented as the mean and standard error (3 repetitions). Matching letters exhibit no significant difference (*p* < 0.01). WSN, water-soluble nitrogen.

**Table 3:** pH and protein content of Gouda water extract.

Sample	pH (2% in Distilled Water)	Proteins (mg BSAE/g DM)
YG	5.69 ± 0.02 ^a^	20.01 ± 0.55 ^c^
MG	6.01 ± 0.01 ^b^	20.12 ± 1.31 ^c^
EG	5.72 ± 0.03 ^a^	12.09 ± 0.41 ^d^

Note: Each result is shown as the mean and standard error (3 repetitions). Matching letters exhibit no significant difference (*p* < 0.01). BSAE, bovine serum albumin equivalent; DM, dry matter; YG, young Gouda extract; MG, medium Gouda extract; EG, extra-sharp Gouda extract.

**Table 4:** Discovery of bioactive peptides in young Gouda extract.

Peptide	Protein Description	Intervals	Expected *m*/*z*	Found at *m*/*z*	Activity	Reference
IQP	αs2-CN	209–211	357.2132	357.2142	ACE inhibition	Jing P. et al., 2014 [22]
LQP	β-CN	103–105	357.2132	357.2142	ACE inhibition	Tonouchi H. et al., 2008 [23]

Note: ACE, angiotensin-converting enzyme.

**Table 5:** Discovery of bioactive peptides in medium Gouda extract.

Peptide	Protein Description	Intervals	Expected *m*/*z*	Found at *m*/*z*	Activity	Reference
EL	αs1-CN	54–55, 156–157, 163–164	261.1445	261.1444	Antioxidant	Suetsuna K. et al., 2000 [24]
IVP	αs1-CN	86–88, 126–128	328.2231	328.2228	ACE inhibition	Jing P. et al., 2014 [22]
VP	β-CN	23–24, 99–100, 188–189, 193–194	215.1390	215.1388	ACE inhibition	Norris R. et al., 2014 [25]
LPP	β-CN	166–168	326.2074	326.2073	ACE inhibition	Norris R. et al., 2014 [25]
VIP	αs2-CN	215–217	328.2231	328.2228	ACE inhibition	Jing P. et al., 2014 [22]
IPP	κ-CN	129–131	326.2074	326.2073	Antioxidant	Chakrabarti S. et al., 2017 [26]
					Anti-inflammatory	Adams C. et al., 2020 [27]
					ACE inhibition	Adams C. et al., 2020 [27]
VPP	β-CN	99–101	312.1918	312.1919	Antioxidant	Chakrabarti S. et al., 2017 [26]
					Anti-inflammatory	Adams C. et al., 2020 [27]
					ACE inhibition	Adams C. et al., 2020 [27]
VVPP	β-CN	98–101	411.2602	411.2600	ACE inhibition	Wang Z-L. et al., 2011 [28]

Note: ACE, angiotensin-converting enzyme.

**Table 6:** Discovery of bioactive peptides in extra-sharp Gouda extract.

Peptide	Protein Description	Intervals	Expected *m*/*z*	Found at *m*/*z*	Activity	Reference
EL	αs1-CN	54–55, 156–157, 163–164	261.1445	261.1447	Antioxidant	Suetsuna K. et al., 2000 [24]
YL	αs1-CN	106–107, 109–110	295.1652	295.1653	ACE inhibition	Mullally M. et al., 1996 [29]
VP	β-CN	23–24, 99–100, 188–189, 193–194	215.1390	215.1382	ACE inhibition	Norris R. et al., 2014 [25]
IR	β-LG	163–164	288.2030	288.2038	ACE inhibition	Murakami, M. et al., 2004 [30]
YPEL	αs1-CN	161–164	521.2606	521.2602	Antioxidant	Suetsuna K. et al., 2000 [24]
DKIHPF	β-CN	62–67	756.4039	756.4045	ACE inhibition	Gobbetti M. et al., 2000 [31]

Note: ACE, angiotensin-converting enzyme.

**Table 7:** ACE inhibition of Gouda water extracts.

Compound (25 mg)	ACE Inhibition Rate (%)
Captopril (12.5 mg)	95.83 ± 0.24
YG	47.02 ± 0.02 ^a^
MG	79.76 ± 0.05 ^b^
EG	94.50 ± 0.05 ^c^

Note: ACE, angiotensin-converting enzyme; YG, young Gouda extract; MG, medium Gouda extract; EG, extra-sharp Gouda extract; Data represented by the mean and standard error (3 repetitions). Matching letters exhibit no significant difference (*p* < 0.01).

## Data Availability

The original contributions presented in the study are included in the article; further inquiries can be directed to the corresponding author.
